# The impact of the war in Ukraine on the prevalence of MDR/RR-TB in Poland

**DOI:** 10.5588/ijtldopen.24.0357

**Published:** 2025-01-01

**Authors:** A. Nowinski, E. Augustynowicz-Kopeć, J. Garnczarek, A. Halicka, M. Koszela, W. Litwiniuk, D. Maj, I. Mazur, J. Niestrój-Ostrowska, R. Podlasin, A. Regulska, M. Wielopolska, J. Wyrwiński, S. Wesołowski, M. Korzeniewska-Koseła

**Affiliations:** ^1^ Department of Tuberculosis Epidemiology and Surveillance, National Tuberculosis and Lung Diseases Research Institute, Warsaw, Poland;; ^2^ Department of Microbiology, National Tuberculosis and Lung Diseases Research Institute, Warsaw, Poland;; ^3^ Second Lung Disease Department in Tuszyn, Center for the Treatment of Lung Diseases and Rehabilitation, Lodz, Poland;; ^4^ Wielkopolskie Center of Pulmonology and Thoracic Surgery, Poznan, Poland;; ^5^ Lower Silesian Oncology, Pulmonology and Hematology Center, Wrocław, Poland;; ^6^ Department of Tuberculosis and Lung Diseases Treatment, Kuyavian-Pomeranian Pulmonology Centre, Bydgoszcz, Poland;; ^7^ Independent Public Tuberculosis and Lung Diseases Sanatorium, Tuberculosis and Lung Diseases Department, Poniatowa, Poland;; ^8^ Małopolski Hospital for Lung Diseases and Rehabilitation, Jaroszowiec, Poland;; ^9^ Hospital for Lung Diseases in Pilchowice, Pilchowice, Poland;; ^10^ Fourth Department, Hospital for Infectious Diseases, Warsaw, Poland;; ^11^ Collegium Medicum, Cardinal Stefan Wyszyński University in Warsaw, Poland;; ^12^ Hospital for Lung Diseases and Long-Term Care, Górno, Poland;; ^13^ Pediatric Department, Mazovian Center for Lung Diseases and Tuberculosis, Otwock, Poland;; ^14^ Independent Public Provincial Hospital, Department I of Tuberculosis and Lung Diseases, Szczecin Zdunowo, Poland.

**Keywords:** Poland, Ukraine, MDR/RR-TB, MDR TB, war, refugees

## Abstract

**BACKGROUND:**

The 2022 invasion of Ukraine by the Russian Federation triggered a refugee crisis, affecting the multidrug-/rifampicin-resistant TB (MDR/RR-TB) prevalence in neighbouring countries. This study examines the epidemiological trends and characteristics of MDR/RR-TB patients in Poland, focusing on the relative contribution of Ukrainian refugees.

**METHODS:**

Data from the Polish National Tuberculosis Registry and EPIC Project database, covering MDR/RR-TB cases reported between 2010 and Q1 2024, were analysed.

**RESULTS:**

The study included 794 MDR/RR-TB cases, showing a demographic shift post-2022. During the 10-year period up to 2021, a median of 48 MDR/RR-TB cases were reported annually in Poland. After 2022, these numbers doubled: 104 cases were reported in 2022 and 101 cases in 2023. Simultaneously, the number of Ukrainian MDR/RR-TB patients increased from 77 (13%) during 2010–2021 to 127 (58%) from 2022 to Q1 2024.

**CONCLUSION:**

Poland is observing an increased number of cases of MDR/RR-TB associated with the large number of displaced Ukrainian citizens who are now residing in Poland. There is a need to monitor the epidemiology of MDR/RR-TB and seek optimal screening and management strategies for TB among refugees from countries with high MDR/RR-TB incidence in Poland and Europe.

## RÉSUMÉ

**CONTEXTE:**

L'invasion de l'Ukraine par la Fédération de Russie en 2022 a engendré une crise des réfugiés, entraînant des conséquences sur la prévalence de la TB multirésistante à la rifampicine (MDR/RR-TB) dans les pays limitrophes. Cette recherche analyse les tendances épidémiologiques ainsi que les caractéristiques des patients atteints de MDR/RR-TB en Pologne, en mettant l'accent sur l'impact des réfugiés ukrainiens.

**MÉTHODES:**

Les informations provenant du Registre National polonais de la TB ainsi que de la base de données du projet EPIC, englobant les cas de MDR/RR-TB déclarés entre 2010 et TB 2024, ont fait l'objet d'une analyse.

**RÉSULTATS:**

L'étude a porté sur 794 cas de MDR/RR-TB, mettant en évidence une évolution démographique après 2022. Sur une période de dix ans jusqu'en 2021, la Pologne a enregistré une médiane de 48 cas de MDR/RR-TB par an. Cependant, après 2022, ces chiffres ont connu une augmentation significative, avec 104 cas signalés en 2022 et 101 en 2023. Parallèlement, le nombre de patients ukrainiens atteints de MDR/RR-TB a augmenté, passant de 77 (13%) entre 2010 et 2021 à 127 (58%) entre 2022 et 2024.

**CONCLUSION:**

La Pologne constate une hausse des cas de MDR/RR-TB, en lien avec l'afflux important de citoyens ukrainiens déplacés sur son territoire. Il est impératif de suivre l'évolution épidémiologique de la MDR/RR-TB et d'explorer des approches efficaces pour le dépistage et la prise en charge de la tuberculose chez les réfugiés provenant de pays où l'incidence de la MDR/RR-TB est élevée, tant en Pologne qu'en Europe.

The invasion of Ukraine by the Russian Federation has precipitated a refugee and humanitarian crisis in Europe. In 2022, the WHO estimated TB incidence in Ukraine was 90/100,000 population,^1^ compared to an estimated 12/100,000 in Poland.^1^ According to a current WHO report, Ukraine is considered a country with a high burden of multidrug- or rifampicin-resistant TB (MDR/RR-TB), with 29% among new and 43% among previously treated and 3,909 laboratory-confirmed MDR/RR-TB cases.^1^ Since the beginning of the war, over 6 million refugees, predominantly women, children, and men over 65 years of age, from Ukraine have been recorded across Europe, of which almost 1 million remained in Poland, according to data from August 2024.^2^ Poland’s borders remain open to incoming people from Ukraine. In 2022, cross-border traffic across the Polish-Ukrainian border increased compared to 2021.^3^ Since the beginning of the war in 2022, there have been over 21 million border crossings.^2,4^

Poland is on the way towards pre-elimination of TB, decreasing the incidence of TB from 18 to 10/100,000 between 2016 and 2021. MDR/RR-TB was diagnosed in 1.1% of new cases and 3.8% of previously treated cases in Poland (2021),^1,5^ with treatment strategies predominantly focused on inpatient care at specialised facilities.

After the beginning of the war in Ukraine, the Polish Ministry of Health (MoH), the National Tuberculosis and Lung Diseases Research Institute (NTLDRI), in cooperation with the WHO and Medicines Sans Frontiers (MSF) launched a joint comprehensive Project ‘MDR-TB Emergency People-centered Response in Poland (EPIC)’ aimed at ensuring quality and free of charge diagnosis, treatment, and care of MDR/RR-TB at outpatient settings, the introduction of digital health solution to improve adherence to treatment and scale up targeted active case finding among the population at high risk for TB.^6^ As a part of the project, several methods to improve TB case detection have been introduced, including educational and informational campaigns for general practitioners and the private sector, covering TB symptoms, referral strategies, and management approaches, as well as establishing a telephone hotline for refugees. The MoH has prepared a legislative framework (pilot programme) to ensure the continuation of TB and DR-TB treatment with the same regimens as used in Ukraine and to promote outpatient treatment. All MDR/RR-TB patients were eligible for the EPIC programme regardless of nationality.

The purpose of this study is to describe the current status of MDR/RR-TB in Poland, with a specific focus on the impact of refugees from Ukraine on the epidemiological landscape.

## METHODS

### Study design and population

This retrospective analysis of the national cohort study utilised data from the National Tuberculosis Registry (NTR) and EPIC Project database managed by the National Tuberculosis and Lung Diseases Research Institute in Warsaw, Poland. The study population comprised patients with MDR/RR-TB who reported between 2010 and Q1 2024 (*n* = 794). The completeness of the data and the potential bias related to the under or overreporting of Ukrainian migrants were checked in the standard way by comparing data from the national TB laboratory network with data reported in the national TB register,^7^ and by further comparison with anonymised data from the EPIC project database. No bias was detected. Descriptive statistics were presented by year of registration, nationality/citizenship, participation in EPIC project, and treatment scheme long vs BPaLM/BPaL (a combination of four drugs: bedaquiline, pretomanid, linezolid, and moxifloxacin [BpaLM] or a combination of three drugs: bedaquiline, pretomanid, and linezolid [BPaL]). Pearson’s χ^2^ test or Fisher’s Exact test was performed to compare categorical variables between groups. Wilcoxon rank-sum test was performed to compare continuous variables between two groups. Polish population counts by sex, age group, and year were downloaded from the Local Data Bank on the Central Statistical Office webpage on 13 June 2024.^8^ All calculations were performed using R v4.2.1 (R Computing, Vienna, Austria).

The study assessed anonymous data from the national surveillance unit and anonymised data from the EPIC project, with approval obtained from the Ethics Committee of the Institute of Tuberculosis and Lung Diseases, Warsaw, Poland (approval number KB-23/2024).

## RESULTS

### Characteristics of the study group

The demographic and clinical data of MDR/RR-TB patients are shown in the Table and the Supplementary Table S1. Demographics and clinical data of MDR-TB patients were compared by period: before (2010–2021) and during the full-scale war in Ukraine (2022–2024 Q1). Data fully reported until April 2024 were included. The dataset comprises 794 cases, 576 from the earlier period and 218 from the latter.

Before the war in Ukraine, during the period 2010–2021, the median number of MDR/RR-TB cases in Poland was 48 per year. After 2022, the number of MDR/RR-TB cases in Poland doubled. In 2022, the number of MDR/RR-TB patients was 104; in 2023, it was 101 (Table).

**Table.** Characteristics of patients with MDR/RR-TB by year of notification (2010–2021 vs. 2022–2024).

Characteristics	Overall, (*n* = 794) *n* (%)	2010–2021 (*n* = 576) *n* (%)	2022–Q1 2024 (*n* = 218) *n* (%)	*P*-value^*^
Annual number of cases, median		48.0	96.9	<0.001
Sex				0.3
Male	612 (77)	449 (78)	163 (75)	
Female	182 (23)	127 (22)	55 (25)	
Age, years				<0.001
Mean ± SD	47 ± 14.8	48 ± 14.9	43 ± 13.7	
Median [IQR]	46.0 [36.0–57.0]	49.0 [37.0–59.0]	42.0 [34.0–50.0]	
Range	1.0–88	1.0–88	4.0–83	
Adult/underage				0.080
Adult	783 (99)	571 (99)	212 (97)	
Underage	11 (1.4)	5 (0.9)	6 (2.8)	
Age category, years				<0.001
0–4	2 (0.3)	1 (0.2)	1 (0.5)	
5–17	9 (1.1)	4 (0.7)	5 (2.3)	
18–39	244 (31)	158 (27)	86 (39)	
40–64	447 (56)	337 (59)	110 (50)	
≥65	92 (12)	76 (13)	16 (7.3)	
Nationality/citizenship				
Poland	530 (67)	458 (80)	72 (33)	
Ukraine	204 (26)	77 (13)	127 (58)	
Armenia	1 (0.1)	0 (0)	1 (0.5)	
Belarus	2 (0.3)	2 (0.3)	0 (0)	
Chechnya	5 (0.6)	5 (0.9)	0 (0)	
Georgia	9 (1.1)	4 (0.7)	5 (2.3)	
India	2 (0.3)	1 (0.2)	1 (0.5)	
Indonesia	1 (0.1)	0 (0)	1 (0.5)	
Kazakhstan	1 (0.1)	1 (0.2)	0 (0)	
Korea	2 (0.3)	2 (0.3)	0 (0)	
Lithuania	1 (0.1)	1 (0.2)	0 (0)	
Moldova	6 (0.8)	3 (0.5)	3 (1.4)	
Nepal	1 (0.1)	0 (0)	1 (0.5)	
Romania	1 (0.1)	0 (0)	1 (0.5)	
Russia	5 (0.6)	2 (0.3)	3 (1.4)	
Turkey	1 (0.1)	1 (0.2)	0 (0)	
Uzbekistan	2 (0.3)	0 (0)	2 (0.9)	
Vietnam	3 (0.4)	3 (0.5)	0 (0)	
Zambia	1 (0.1)	1 (0.2)	0 (0)	
Other	16 (2.0)	15 (2.6)	1 (0.5)	
Nationality				<0.001
Poland	530 (67)	458 (80)	72 (33)	
Ukraine	204 (26)	77 (13)	127 (58)	
Other	60 (7.6)	41 (7.1)	19 (8.7)	
Patient participates in the EPIC Project				>0.9
No	N/A	N/A	68 (31)	
Yes	N/A	N/A	150 (69)	
Treatment scheme				<0.001
Local standard	619 (79)	559 (97)	60 (29)	
WHO long regiment	110 (14)	16 (3.0)	95 (45)	
BPaLM/BPaL	54 (7.0)	N/A	54 (26)	
Unknown	10	1	9	
TB classification				0.4
Pulmonary	771 (97)	561 (97)	210 (96)	
Extrapulmonary	23 (2.9)	15 (2.6)	8 (3.7)	
Relapse				0.025
No	527 (66)	369 (64)	158 (72)	
Yes	267 (34)	207 (36)	60 (28)	
Comorbidities: HIV/AIDS				0.2
No	125 (15.7)	14 (2.4)	111 (50.9)	
Yes	32 (4.0)	1 (0.2)	31 (14.3)	
Unknown	637 (80.3)	561 (97.4)	76 (34.8)	

* Pearson's χ^2^ test; Wilcoxon rank sum test; Fisher's exact test.

MDR/RR-TB = multidrug-resistant/rifampicin-resistant TB; SD = standard deviation; IQR = interquartile range; EPIC = Emergency People Centered Response Program; BpaLM = a combination of four drugs: bedaquiline, pretomanid, linezolid, and moxifloxacin; N/A = not available.

The sex distribution shows 612 males (77% overall) and remained constant over time. Age analysis reveals a median age of 49 years from 2010 to 2021. This median significantly decreased to 42 years later, indicating a younger demographic in the recent data. (*P* < 0.001).

The categorisation of people into adults and minors reveals that adults dominate the group, comprising 99% of the entire group. Age categories reflect notable shifts: the predominant 40-64 age group decreased from 59% to 50%, while the 18–39-years age group increased from 27% to 39% (*P* < 0.001).

**Figure 1.**
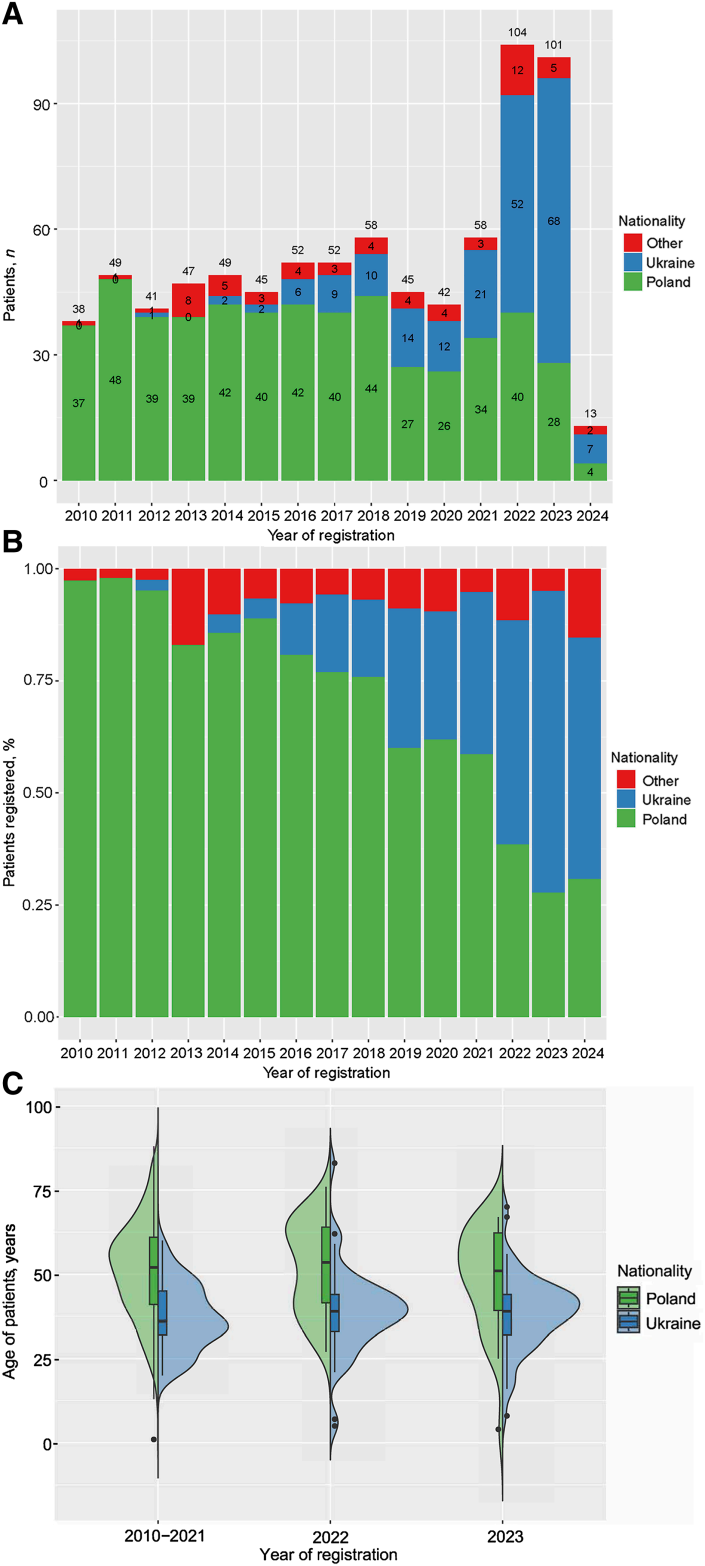
**A)** Number of patients by nationality/citizenship by year of registration. Data from 2024 only covers the first quarter. **B)** Proportion of patients by nationality/citizenship by year of notification/registration. Data from 2024 only covers the first quarter. **C)** Age distribution by nationality/citizenship across the three periods analysed.

Nationality distribution also showed significant changes, particularly with a decrease in Polish patients from 80% to 33% and an increase in Ukrainian patients from 13% to 58% (Figure 1A and 1B) (*P* < 0.001), which influenced the previously described change in the age distribution (Figure 1C).

Since mid-2022, patients have been enrolled in the EPIC project, and throughout the period from 2022 to Q1 2024, 67% of the patients in the country were covered by EPIC care, leading to a rapid change in the management of MDR-TB treatment. Treatment schemes evolved significantly, with hospital local treatment standards decreasing from 97% to 29% and longer WHO-regimen treatments and BPaLM/ BPaL therapy increasing notably (*P* < 0.001).

The most common cases were those of pulmonary TB. There were no differences in the relapse rates between both groups. In the group of patients treated since 2022, we observed 22% of patients living with HIV. However, comparing this group with the earlier one is challenging because we only have incomplete data regarding the coexisting diseases from 2010 to 2021.

**Figure 2.**
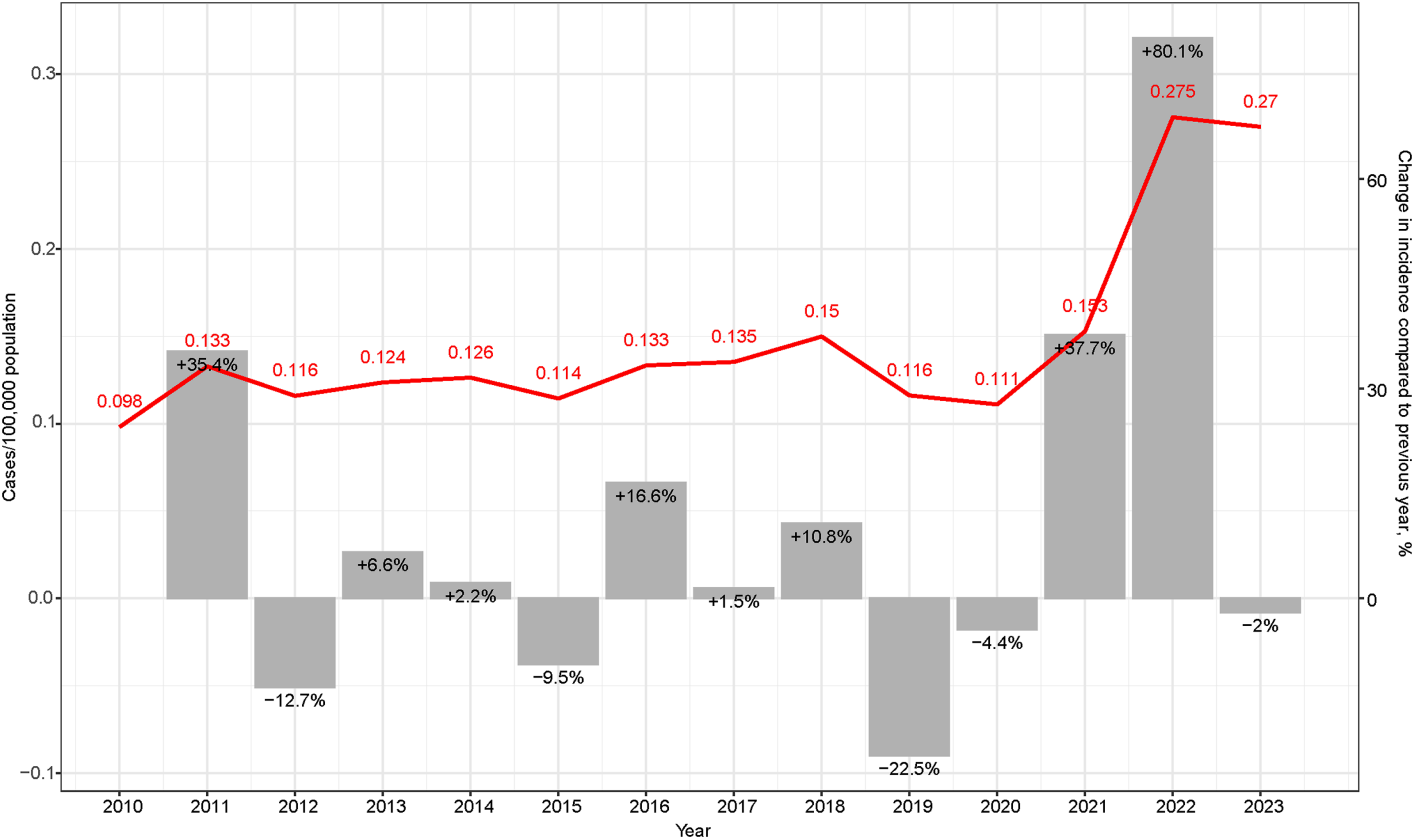
Incidence and year-to-year percentage changes in the number of MDR/RR-TB cases standardised by sex and age in Poland, 2010–2022. MDR/RR-TB = multidrug-resistant/rifampicin-resistant TB.

**Figure 3.**
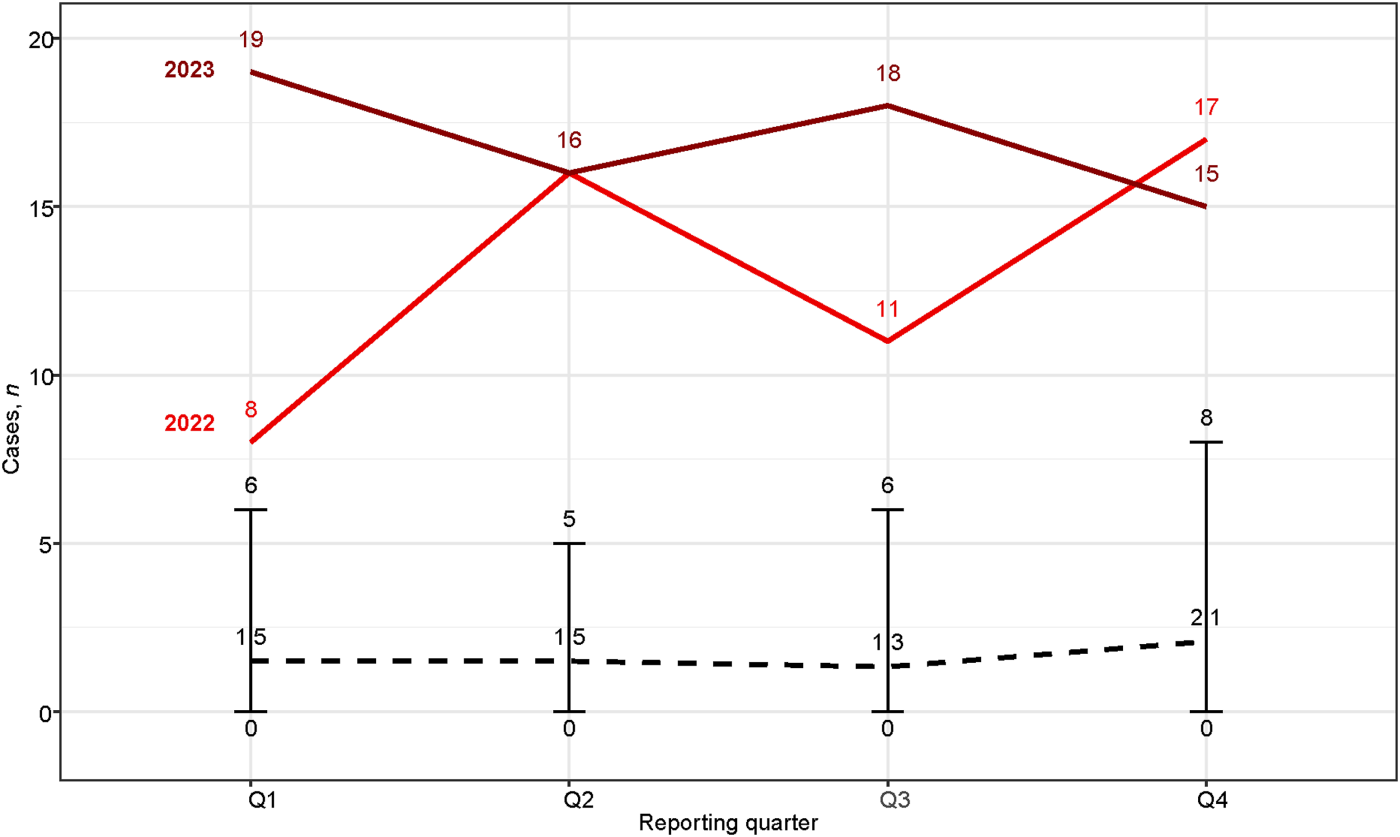
Quarterly notifications of MDR/RR-TB cases among individuals with Ukrainian nationality/citizenship and Poland in 2022 and 2023, compared with pooled data from 2010 to 2021. Light red solid line represents quarterly Ukrainian nationality/citizenship of MDR-TB cases in 2022. Dark red solid line represents quarterly Ukrainian nationality/citizenship of MDR-TB cases in 2023. Black dashed line with bars represents mean, minimum and maximum quarterly Ukrainian nationality/citizenship number of MDR-TB cases in 2010–2021. MDR/RR-TB = multidrug-resistant/rifampicin-resistant TB.

The incidence rate of MDR-TB cases per 100,000 population showed a significant increase in 2022 (Figure 2), similar to the marked rise observed in the monthly number of reported patients (Figure 3). In addition, the number and proportion of Ukrainian cases increased in 2023 compared to 2022 (Figures 1 and 2).

**Figure 4.**
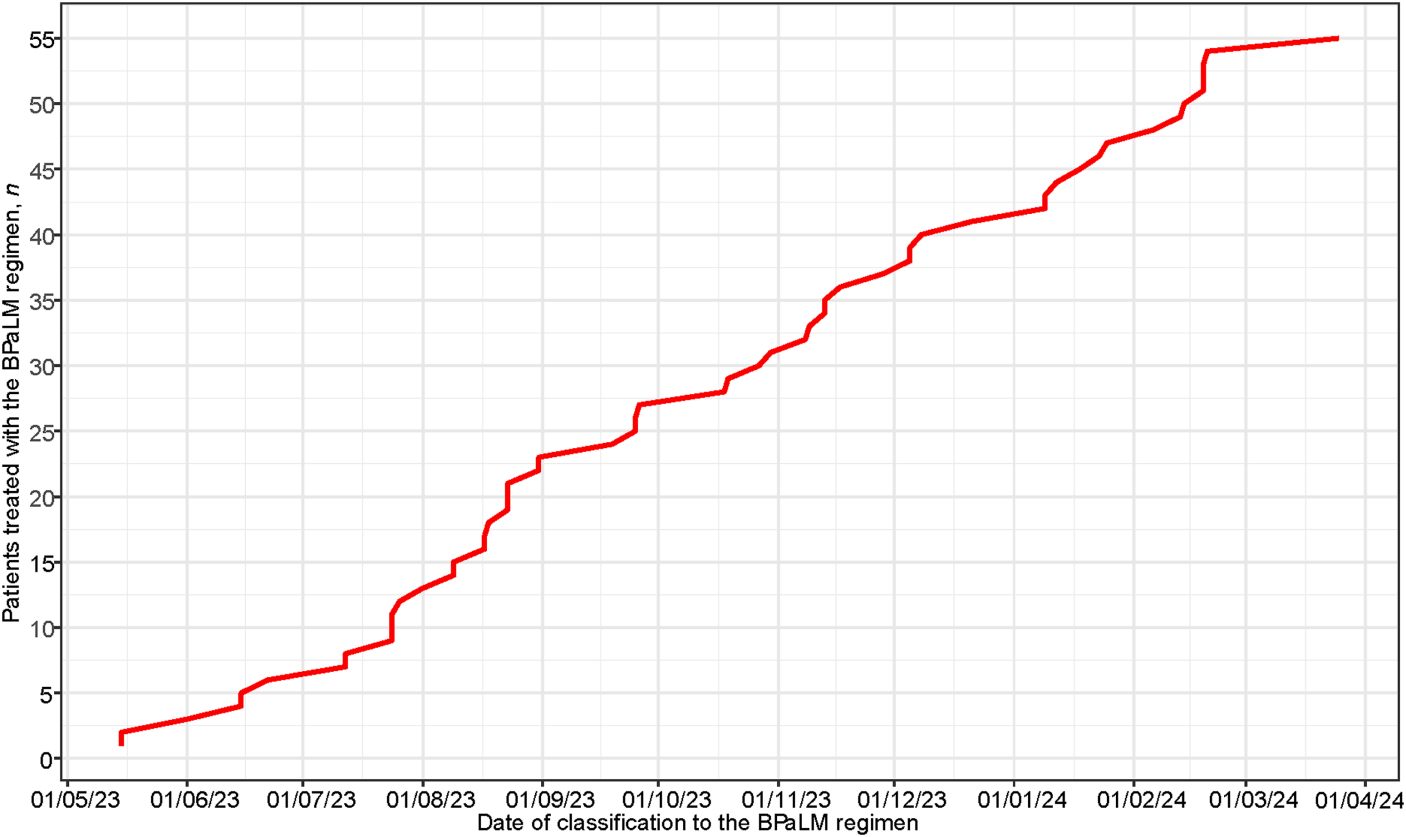
Cumulative number of patients treated with the BPaLM/BPaL regimen. BpaLM = a combination of four drugs: bedaquiline, pretomanid, linezolid, and moxifloxacin; BPaL = a combination of three drugs: bedaquiline, pretomanid, and linezolid.

By the time we analysed the data for this publication, 55 patients had started treatment with the BPaLM/BPaL regimen. The cumulative graph of new patients being enrolled in BPaLM/BPaL treatment as soon as WHO recommendations allowed is shown in Figure 4.

## DISCUSSION

We have observed an increase in the number of MDR/RR-TB cases in Poland since 2022, coinciding with the onset of the full-scale war in Ukraine. In 2022 and 2023, these cases exceeded 100 notifications per year, which is a two-fold increase compared to the 10-year mean incidence, which was 48 (standard deviation [SD] 6.3) in 2010–2021. In 2022, in Poland, the age-standardised incidence rate of MDR/RR-TB was 0.275/100,000, representing an 80% increase compared to the previous year. This result aligns with WHO estimates, which predicted an increase in MDR/RR-TB cases due to the movement of refugees from Ukraine.^9^

However, we still observed fewer cases of MDR/RR-TB in Poland than estimated by the WHO Euro calculator.^9^ From the beginning of 2022 to the first quarter of 2024, 218 cases of MDR/RR-TB were recorded in Poland, including individuals born in Poland and in other countries.

In addition, we observed that both the number and proportion of Ukrainian MDR/RR-TB cases increased in 2022 and 2023 compared to previous years (Figures 1 and 2). To evaluate our data, referring to other data from the region is necessary. To date, aggregate data have been presented,^10^ and several European countries have submitted detailed reports. There have been reports from national registries of Poland’s neighbours. In the work of Hauer et al., it was found that the wave of refugees from Ukraine has impacted the frequency of TB notifications in Germany.^11^ What is particularly interesting from a Polish perspective is that a similar wave was observed in Germany during the previous refugee wave in 2015 when a wave of refugees from the Middle East reached Germany. Czechia also observed the impact of refugees from Ukraine on the frequency of TB detection in the country.^12^ German studies also discussed the number of cases detected, referring to possible numbers of cases of people from Ukraine who reached Europe after the war and the actual cases registered. Similar to our study, Hauer et al. found a lower number of cases than estimated by the WHO.

The reason for the discrepancy between epidemiological estimates and case notifications was widely discussed and may depend on the TB detection model adopted by the country. Published data from TB screenings in France,^13^ Wales,^14^ Germany,^15^ are quite challenging to analyse due to the different screening methods proposed by various countries. De Vries et al. gathered epidemiological data from across Europe, finding that the total number of TB cases among refugees from Ukraine in Europe is variable and seems to depend on the screening model for TB adopted in the country. Countries with long-standing traditions of screening migrants and refugees arriving in their countries, such as Norway, have a higher percentage of individuals diagnosed with TB than those that do not screen the entire refugee populations, such as Poland, among others.^10^

According to the WHO and European Centre for Disease Prevention and Control (ECDC) recommendation, mandatory screening for TB and testing for TB of the entire refugee population is not recommended and, thus, has not been applied in Poland.^16^ It is necessary to consider previous studies that present various solutions (e.g., targeted screening)^17–19^ and experiences from the past^20,21^ to properly plan assistance for refugees in the context of TB diagnosis and treatment.

Screening migrants for TB has been conducted in some European countries for years,^22,23^ but not in Poland. This is mainly due to historical conditions in which Western European countries typically received migrants, while Eastern European countries were more often a source of migration.^24^ Recent economic changes and the economic growth of Central and Eastern European countries have made these countries, including Poland, a migration destination, posing new challenges for healthcare systems. In the authors’ opinion, based on the experiences of the EPIC project, the most beneficial approach in Poland currently seems to be the implementation of an expanded targeted screening system for TB in patients of primary care and occupational medicine. Such a targeted screening system should include individuals from groups at risk of TB as well as those migrating to Poland from countries with high TB incidence rates. However, no decisions have been made in Poland regarding the level of incidence in the countries from which refugees and migrants arrive that should be considered as the threshold for recommending screening tests.

Our data highlights demographic shifts: the predominant 40–64-years age group decreased from 59% to 50%, while the 18–39-years age group increased due to the influx of younger refugees. These results correlate with the demographics of Ukraine, where a slightly younger population is affected by TB compared to Poland.^1^

Until 2022, Polish hospitals used local, hospital-individualised treatment regimens. Since the introduction of the EPIC project, standardised treatment regimens in accordance with WHO standards have been widely implemented. When recommendations for the BPaLM/BPaL treatment regimen emerged, BPaLM/BPaL treatment was implemented as an EPIC standard procedure in outpatient settings, along with video-supported treatment monitoring. When we analysed the data for this publication, most MDR/RR-TB patients in Poland were receiving treatment with the BPaLM/BPaL as the first-choice treatment.

In the first quarter of 2024, 77% of newly diagnosed MDR/RR-TB patients started treatment with the BPaLM/BPaL regimen (Supplementary Table S2). The authors believe this is an important outcome of the EPIC programme because access to the BPaLM/BPaL regimen varies across Europe, as shown in several publications.^25,26^

The data we presented are not entirely satisfactory. Due to the lack of accurate data on HIV infections in the population of Polish TB patients, we cannot address the potential increase in the percentage of individuals with HIV-TB coinfection. Additionally, despite our efforts, we have not yet obtained reliable data on cross-border treatment, i.e., situations where TB symptoms appear while staying in Poland, but the patient (usually women) may return to their home country (e.g., Ukraine) for diagnosis and treatment.

In this publication, we used citizenship to assess the country of origin. This is the traditional approach in the TB register in Poland, as until recently, the relatively small number of migrants from Ukraine applying for Polish citizenship reflected nationality quite well. Another element that needs to be considered when evaluating data from 2024 is the possible delay in data notification. Considering the structure of the Polish TB registry, which relies on paper forms sent by mail, data from the first quarter of 2024 may be incomplete and should be estimated accordingly.

## CONCLUSIONS

Poland is observing an increased number of cases of MDR/RR-TB associated with the large number of displaced Ukrainian citizens who are now residing in Poland. There is a need to monitor the epidemiology of MDR/RR-TB and seek optimal screening and management strategies for TB among refugees from countries with high MDR/RR-TB incidence in Poland and Europe.

## Supplementary Material


