# Supplementary material for: The impact of the war in Ukraine on the prevalence of MDR/RR-TB in Poland
Source: IJTLD Open. 2025 Jan 1;2(1):6–12. doi: 10.5588/ijtldopen.24.0357 (PMC11724529; doi:10.5588/ijtldopen.24.0357)
Supplement: Supplementary file 1 [file ijtldopen24-0357_supplementarydata1.docx]

# The impact of the war in Ukraine on the prevalence of MDR/RR-TB in Poland

Supplementary Table S1

Multidrug-resistant tuberculosis - statistical analysis

All data - by year of registration (2010-2021 vs. 2022 vs. 2023 vs. 2024)

| **Multidrug-resistant tuberculosis - descriptive statistics - all data - by year of registration** | **Overall,**  **N = 794^1^** | **2010-2021,**  **N = 576^1^** | **2022,**  **N = 104^1^** | **2023,**  **N = 101^1^** | **Q1 2024,**  **N = 13^1^** | **p-value^2^** |
| --- | --- | --- | --- | --- | --- | --- |
| Gender |  |  |  |  |  | 0.5 |
| male | 612 (77%) | 449 (78%) | 81 (78%) | 73 (72%) | 9 (69%) |  |
| female | 182 (23%) | 127 (22%) | 23 (22%) | 28 (28%) | 4 (31%) |  |
| Age |  |  |  |  |  | <0.001 |
| Mean (SD) | 47 (14.8) | 48 (14.9) | 44 (14.4) | 42 (13.5) | 40 (9.6) |  |
| Median [IQR] | 46.0 [36.0, 57.0] | 49.0 [37.0, 59.0] | 42.0 [35.0, 53.2] | 42.0 [34.0, 49.0] | 41.0 [34.0, 43.0] |  |
| Range | 1.0, 88 | 1.0, 88 | 5.0, 83 | 4.0, 81 | 20.0, 58 |  |
| Adult/underage |  |  |  |  |  | 0.070 |
| adult | 783 (99%) | 571 (99%) | 102 (98%) | 97 (96%) | 13 (100%) |  |
| underage | 11 (1.4%) | 5 (0.9%) | 2 (1.9%) | 4 (4.0%) | 0 (0%) |  |
| Age category |  |  |  |  |  |  |
| 0-4 | 2 (0.3%) | 1 (0.2%) | 0 (0%) | 1 (1.0%) | 0 (0%) |  |
| 5-17 | 9 (1.1%) | 4 (0.7%) | 2 (1.9%) | 3 (3.0%) | 0 (0%) |  |
| 18-39 | 244 (31%) | 158 (27%) | 41 (39%) | 40 (40%) | 5 (38%) |  |
| 40-64 | 447 (56%) | 337 (59%) | 52 (50%) | 50 (50%) | 8 (62%) |  |
| 65+ | 92 (12%) | 76 (13%) | 9 (8.7%) | 7 (6.9%) | 0 (0%) |  |
| Nationality/Citizenship |  |  |  |  |  |  |
| Poland | 530 (67%) | 458 (80%) | 40 (38%) | 28 (28%) | 4 (31%) |  |
| Ukraine | 204 (26%) | 77 (13%) | 52 (50%) | 68 (67%) | 7 (54%) |  |
| Armenia | 1 (0.1%) | 0 (0%) | 1 (1.0%) | 0 (0%) | 0 (0%) |  |
| Belarus | 2 (0.3%) | 2 (0.3%) | 0 (0%) | 0 (0%) | 0 (0%) |  |
| Chechnya | 5 (0.6%) | 5 (0.9%) | 0 (0%) | 0 (0%) | 0 (0%) |  |
| Georgia | 9 (1.1%) | 4 (0.7%) | 3 (2.9%) | 2 (2.0%) | 0 (0%) |  |
| India | 2 (0.3%) | 1 (0.2%) | 0 (0%) | 1 (1.0%) | 0 (0%) |  |
| Indonesia | 1 (0.1%) | 0 (0%) | 1 (1.0%) | 0 (0%) | 0 (0%) |  |
| Kazakhstan | 1 (0.1%) | 1 (0.2%) | 0 (0%) | 0 (0%) | 0 (0%) |  |
| Korea | 2 (0.3%) | 2 (0.3%) | 0 (0%) | 0 (0%) | 0 (0%) |  |
| Lithuania | 1 (0.1%) | 1 (0.2%) | 0 (0%) | 0 (0%) | 0 (0%) |  |
| Moldova | 6 (0.8%) | 3 (0.5%) | 1 (1.0%) | 0 (0%) | 2 (15%) |  |
| Nepal | 1 (0.1%) | 0 (0%) | 1 (1.0%) | 0 (0%) | 0 (0%) |  |
| Romania | 1 (0.1%) | 0 (0%) | 1 (1.0%) | 0 (0%) | 0 (0%) |  |
| Russia | 5 (0.6%) | 2 (0.3%) | 2 (1.9%) | 1 (1.0%) | 0 (0%) |  |
| Turkey | 1 (0.1%) | 1 (0.2%) | 0 (0%) | 0 (0%) | 0 (0%) |  |
| Uzbekistan | 2 (0.3%) | 0 (0%) | 1 (1.0%) | 1 (1.0%) | 0 (0%) |  |
| Vietnam | 3 (0.4%) | 3 (0.5%) | 0 (0%) | 0 (0%) | 0 (0%) |  |
| Zambia | 1 (0.1%) | 1 (0.2%) | 0 (0%) | 0 (0%) | 0 (0%) |  |
| Other | 16 (2.0%) | 15 (2.6%) | 1 (1.0%) | 0 (0%) | 0 (0%) |  |
| Nationality |  |  |  |  |  |  |
| Poland | 530 (67%) | 458 (80%) | 40 (38%) | 28 (28%) | 4 (31%) |  |
| Ukraine | 204 (26%) | 77 (13%) | 52 (50%) | 68 (67%) | 7 (54%) |  |
| Other | 60 (7.6%) | 41 (7.1%) | 12 (12%) | 5 (5.0%) | 2 (15%) |  |
| Treatment scheme |  |  |  |  |  |  |
| Local standard | 619 (79%) | 559 (97%) | 47 (45%) | 13 (14%) | 0 (0%) |  |
| WHO long | 110 (14%) | 15 (2.6%) | 54 (52%) | 38 (41%) | 3 (23%) |  |
| BPALM | 55 (7.0%) | 0 (0.0%) | 3 (2.9%) | 41 (45%) | 10 (77%) |  |
| Unknown | 10 | 1 | 0 | 9 | 0 |  |
| TB classification |  |  |  |  |  | 0.3 |
| pulmonary | 771 (97%) | 561 (97%) | 98 (94%) | 99 (98%) | 13 (100%) |  |
| extrapulmonary | 23 (2.9%) | 15 (2.6%) | 6 (5.8%) | 2 (2.0%) | 0 (0%) |  |
| Relapse |  |  |  |  |  | 0.010 |
| No | 527 (66%) | 369 (64%) | 67 (64%) | 80 (79%) | 11 (85%) |  |
| Yes | 267 (34%) | 207 (36%) | 37 (36%) | 21 (21%) | 2 (15%) |  |
| ^1^n (%) | | | | | | |
| ^2^Fisher's exact test; Kruskal-Wallis rank sum test | | | | | | |
